# Sella turcica measurements on lateral cephalograms of patients with neurofibromatosis type 1

**DOI:** 10.3205/iprs000107

**Published:** 2017-03-23

**Authors:** Reinhard E. Friedrich, Johanna Baumann, Anna Suling, Hannah T. Scheuer, Hanna A. Scheuer

**Affiliations:** 1 Department of Oral and Craniomaxillofacial Surgery, Eppendorf University Hospital, University of Hamburg, Germany; 2 Institute of Medical Biometry and Epidemiology, Eppendorf University Hospital, University of Hamburg, Germany; 3 Department of Orthodontics, Eppendorf University Hospital, University of Hamburg, Germany

**Keywords:** neurofibromatosis type 1, sella turcica, plexiform neurofibroma, trigeminal nerve, cephalometry, skull base surgery, anthropology

## Abstract

The aim of this study was to measure line segments and areas of sella turcica on lateral cephalograms with respect to the clinical diagnosis of facial phenotype of patients with neurofibromatosis type 1 (NF1). Special attention was given to correlate the measured values with certain tumour types that are typical for this disease.

**Material and methods:** Lateral cephalograms of 194 individuals were investigated. Patients with NF1 were further divided according to the detection and topography of facial plexiform neurofibromas (PNF) taking into account the distribution pattern of the trigeminal nerve. All patients with PNF showed unilateral tumour localisation. Patients without any facial PNF constituted a separate group. Healthy volunteers with ideal occlusion and no history of any intervention in the maxillofacial region served as a control group. The following items were determined on the radiographs: sella entrance, sella width, sella depths, sella diagonal, and sella area.

**Results:** Patients with PNF of the first and second trigeminal nerve branch or affected in all branches showed highly statistically significant enlarged sella tucica measurement values. On the other hand, patients with PNF restricted to one branch only or simultaneously in the second and third branches showed measurement values that were not different to those obtained in NF1 patients devoid of facial PNF. The latter group also showed no difference of sella turcica parameters obtained in the control group.

**Conclusion:** This study provides evidence for the association of a certain NF1 phenotype with distinct skeletal alterations of the skull base, shown here using the example of the representation of the sella turcica in the lateral radiograph. These findings are also relevant in the discussion of NF1 as a disease of bones and in the assessment of brain development in NF1. Both items are discussed in relationship to a facial plexiform neurofibroma. Furthermore, the knowledge of this association of findings provides the clinician with relevant information in the planning of skull base procedures in these patients.

## Zusammenfassung

Das Ziel dieser Untersuchungen war die Vermessung von Strecken und Flächen der Sella turcica auf seitlichen Fernröntgenbildern unter besonderer Berücksichtigung des fazialen Phänotyps von Patienten, die an einer Neurofibromatose Typ 1 (NF1) erkrankt waren. Besondere Aufmerksamkeit wurde der Korrelation von definierten Tumortypen, die für diese Erkrankung typisch sind, und den kephalometrischen Messwerten gewidmet.

**Material und Methode:** Laterale Kephalogramme von 194 Individuen wurden untersucht. Patienten mit NF1 wurden weiter differenziert nach dem Nachweis und der Topographie fazialer plexiformer Neurofibrome (PNF), wobei das Verteilungsmuster des N. trigeminus im Gesicht als Orientierung diente. Alle Patienten mit PNF hatten eine einseitige Manifestation des Tumors. Patienten ohne faziales PNF stellten eine gesondert analysierte Gruppe dar. Gesunde Probanden mit Idealokklusion und ohne jede Vorgeschichte einer Intervention im kraniomaxillofazialen Bereich dienten als Kontrollgruppe. Die folgenden Messungen wurden durchgeführt: Sella-Eingang, Sella-Weite, Sella-Tiefe, Sella-Diagonale und Sella-Fläche.

**Ergebnisse:** Patienten mit einem PNF des ersten und zweiten oder solche, die in allen drei Ästen des N. trigeminus von diesem Tumor betroffen sind, weisen statistisch signifikant vergrößerte Sella-Messwerte auf. Andererseits sind die Messwerte der Patienten, die nur in einem einzelnen Ast vom PNF betroffen sind oder deren Tumorausbreitung sich auf den 2. und 3. Ast des Nervs beschränkt, von denen der Patienten ohne ein faziales PNF statistisch nicht zu unterscheiden. Die Messwerte dieser beiden Gruppen unterscheiden sich ebenfalls nicht von denen der Kontrollgruppe.

**Schlussfolgerung:** Diese Untersuchung beweist die Assoziation eines bestimmten Ausbreitungstyps des fazialen PNF bei NF1-Patienten mit merklichen skelettalen Veränderungen der Schädelbasis, hier dargestellt anhand der Form der Sella turcica im seitlichen Röntgenbild. Diese Befunde sind auch bedeutsam für die Diskussion der NF1 als Erkrankung des Skelettsystems und für die Einschätzung der Gehirnentwicklung bei NF1, beide in Zusammenhang mit dem Nachweis eines plexiformen Neurofibroms. Weiterhin liefern die Kenntnisse über die hier vorgestellten Befunde dem Kliniker relevante Informationen für die Planung von Schädelbasiseingriffen an Patienten mit dieser Diagnose.

## Introduction

### Neurofibromatosis type 1

Neurofibromatosis type 1 (NF1) is an autosomal dominant inherited disease [1]. The denomination of NF1 is derived from neurofibroma, a nerve sheath cell tumour usually occurring in this condition in large numbers and preferentially affecting the integument. This term was originally proposed by von Recklinghausen, the pathologist who gave the first complete morphological description of the tumours [2]. At present, NF1 is classified a tumour suppressor gene disease [3]. Many findings of NF1 are explained as a result of loss of tumour suppressor gene function. Neurofibromin is the *NF1* product and functions to suppress tumour development [4], [5]. However, neurofibromin also has other largely unknown functions. For example, neurofibromin is expressed in neural cells during brain development. Furthermore, the effect of modifying genes on the NF1 phenotype is further discussed in the literature [6], [7], [8].

NF1 occurs more frequently than many other rare diseases, in particular in comparison to other neurocutaneous disorders [9]. About one in 2,500 to one in 4,000 live births will be affected with NF1 [10], [11]. Besides the name-giving neurofibromas and a predisposition for developing other benign and malignant neoplasms [12], [13], NF1 is also a disease of bone [14]. Bone can be affected in *general*, e.g. patients are frequently of small stature compared to controls [15] and they are at risk of developing osteoporosis very early in life [16], [17], and also *local*, e.g. pseudarthrosis of long bones [18] and sphenoid wing dysplasia [19] (Table 1 (Tab. 1)). 

### Skull

Localised deformities of the cranio-facial skeleton in NF1 are associated with adjacent facial plexiform neurofibroma (PNF) in the majority of cases [20]. Facial PNFs are provisionally categorised according to the facial sensory innervation fields of the trigeminal nerve [21]. This correlation of skeletal dysplasia and evidence of a certain type of tumour in the context of NF1 was also revealed for sphenoid wing dysplasia [22], a major diagnostic criteria when establishing NF1 diagnosis (Table 1 (Tab. 1)). However, not in every surgical case treated for orbital dysplasia was there also tumour detected at all or at least in proximity to the dysplastic sphenoid [19], [23]. Treatment of the orbitotemporal region in NF1 is frequently required for the reduction of pulsating exophthalmos associated with sphenoid wing dysplasia by means of skeletal reconstruction [24] and relief from severe aesthetic distortions following invasive growth of plexiform neurofibroma [25], [26].

### Sella turcica

**Figure 1:**
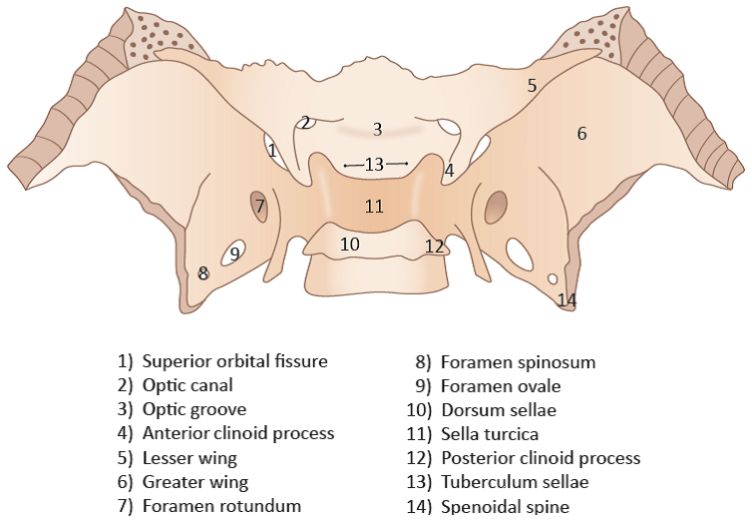
Simplified schematic drawing of the sphenoid bone, dorsal and slightly cranial view. Relevant structures are identified by numbers. The figure shows the proximity of the foramina of the trigeminal nerve to the sella turcica. The branches of the trigeminal nerve pass through the superior orbital fissure ((1), nervus ophthalmicus), foramen rotundum ((7) nervus maxillaris), foramen ovale ((9) nervus mandibularis) and foramen spinosum ((8) ramus meningeus of the mandibular nerve). Tuberculum sellae (13) and dorsum sellae (10) constitute the vertical borders of sella turcica and are landmarks on lateral skull radiograms.

Sella turcica is a key point of lateral cephalometry analysis in patients subjected to orthodontic treatment and also in many fields of skull anthropometry [27] (Figure 1 (Fig. 1)). Sella turcica shows some physiological variations of both size and shape visible on this radiographic projection [28], [29]. However, thorough analysis of sella turcica on cephalograms obtained for orthodontic or craniofacial treatment planning is recommended in order to identify deviations of the normal radiographic appearance that could give clues to local or general pathology [30], [31], [32]. In NF1, deformities of the sella are well recognised findings on lateral plain radiographs [33], [34]. Initially, these deformities – as visualised on plain radiographs – gave rise to neurosurgical interventions that proved no associated pathology [35]. The pathogenesis of sella turcica deformity in NF1 is unknown and this item has been poorly addressed in the literature until now [36]. On the other hand, assessment of pituitary function in these patients is an important diagnostic task [37]. Description of sella turcica morphology on plain radiographs insufficiently correlates with hypothalamic-pituitary malfunctions in many cases [38]. Therefore, the focus of scientific radiographic description of sella turcica morphology on plain radiographs has shifted from assessing localised functional endocrinological disorders and other pathologies towards the characterisation of localised osseous alterations in the context of syndromes [38], [39]. Indeed, the implementation of cross-sectional imaging techniques allow the detailed visualisation of soft and hard tissues and have revealed that no distinct primary tumour pathology of the pituitary is regularly associated with an enlarged sella turcica in NF1 affected individuals [40]. Nevertheless, endocrine disorders are occasionally noted in NF1 [40]. These disorders are usually diagnosed in patients with optic pathway glioma (OPG) [41] and are explained as the consequence of peculiar tumour growth patterns and local pressure of OPG on the hypothalamic-pituitary axis [42], [43]. OPG with intrasellar extension is extremely rare [44] and this also applies to primary intrasellar pilocystic astrocytomas [45]. Genetic causes were suspected for the assessment of cranial abnormalities in NF1 such as those of the skull base. Indeed, a reduction of the anterior-posterior dimension of the skull base in NF1 was explained with reference to a hypothetical haploinsufficient bone in this region [46]. Considering this assumption (derived from cephalometric analysis), the area of the sella turcica may differ from those of healthy individuals. In addition, a congenitally developing facial PNFs originating from the trigeminal nerve origin at the skull base adjacent to the sella turcica could have effects on sella turcica development and area. This impact on sella turcica area could be measurable on lateral cephalograms. Therefore, this study attempted to analyse sella turcica area on radiographs of this type obtained in a group of NF1 affected patients.

## Materials and methods

Lateral cephalometric radiographs of 194 individuals were included in this study (Table 2 (Tab. 2) and Table 3 (Tab. 3)). The radiographs had been performed over a period of 21 years (1989–2009) in the Department of Oral and Craniomaxillofacial Surgery, UKE. Indication for radiography in patients was the survey of relevant skeletal alterations of the skull, possibly associated with disease. All patients had given informed consent for the scientific evaluation of these diagnostic measures. All X-ray documents were anonymous so that only age and gender were known to the investigators at the time of measurement.

### Patient group

The lateral cephalograms of 165 patients with an established NF1 diagnosis [1] were evaluated in this study (age: 4 to 78 years; females: 89, males: 77). The patient group was differentiated according to disease-relevant features of the facial phenotype. Some patients showed small cutaneous neurofibromas all over the body and also in the facial region in most cases, but they had not developed a facial plexiform neurofibroma (PNF). Patients of this phenotype are declared as the group of disseminated-cutaneous neurofibroma (DCNF) patients (irrespective of whether these patients had developed PNF outside the head and neck region). The second group is characterised by the detection of facial PNF (PNF group). Most of these tumours were histologically verified in the context of facial plastic surgery to reduce tumour masses and to alleviate from impaired facial functions (REF). In the facial region, these tumours are diagnosed as invasive diffuse PNF. The patients of the PNF group were further characterised with respect to the affected facial structures (Table 4 (Tab. 4)). The assumed origin of neurofibromas is peripheral nerve sheath cells [1]. Therefore, with respect to facial appearance, the topography of the individual facial PNF extension roughly correlates to the dermatomes of the trigeminal nerve [21], [47]. However, tumour volume is variable in facial PNF patients and also varies the extension of a lesion within a defined trigeminal nerve branch, i.e. the nerve branch that is visibly affected is not necessarily completely tumorous. Furthermore, PNF show unpredictable progression of growth both in size at the site of the established tumour and regarding invasion of adjacent structures. Therefore, the distinction of PNF subgroups with respect to affected branch(es) can show overlaps between adjacent branches and also to sites that do not belong to the territories of the nerve branches of the trigeminal nerve. The most severely affected patients show the involvement of all three trigeminal nerve branches by a PNF which gives the impression of a hemifacial PNF [48]. As a consequence of the phenotypic variations, the subgroups comprise both patients with tumorous disorders of individual nerve branches and also combinations of the nerve branches in cases of dermatome-overlapping tumours. This clinical distinction initially led to the formation of 6 subgroups. After the first analysis of the measured values, the combination of PNF patients with four main groups proved to be useful to adequately describe differences and coincidences of sella turcica measurement values with respect to phenotypes (Table 4 (Tab. 4)). One further analysis was carried out to delineate sella turcica measurement values with respect to the facial level of PNF (Table 4 (Tab. 4)). 

### Control group

The reference group consisted of 29 individuals (17 males (58.62%), 12 females (41.38%). The age of these participants ranged from 17 to 26 years in females (mean: 23.2 years) and 16 to 35 years in males (mean: 25.7 years). This reference group was a collection of well-defined lateral cephalograms of individuals that had voluntarily contributed their radiographs to a former study [49], [50]. These subjects all exhibited ideal dental occlusion, without ever having been treated by orthodontics or any history of trauma or craniofacial malformation. We chose this archival group for comparison in order to define the sella turcica area in individuals without any deviation from ideal occlusal parameters. Recent studies have pointed to the association of sella turcica morphology and disproportions of jaws [51]. Basic principles of the cephalometric analysis of this study are detailed elsewhere [52]. A second reason to use an archival group with characteristics of ideal occlusion was the insight that a radiation exposure of suitable candidates for a control group of this kind is prohibited for ethical reasons.

### Data registration and measurement

**Figure 2:**
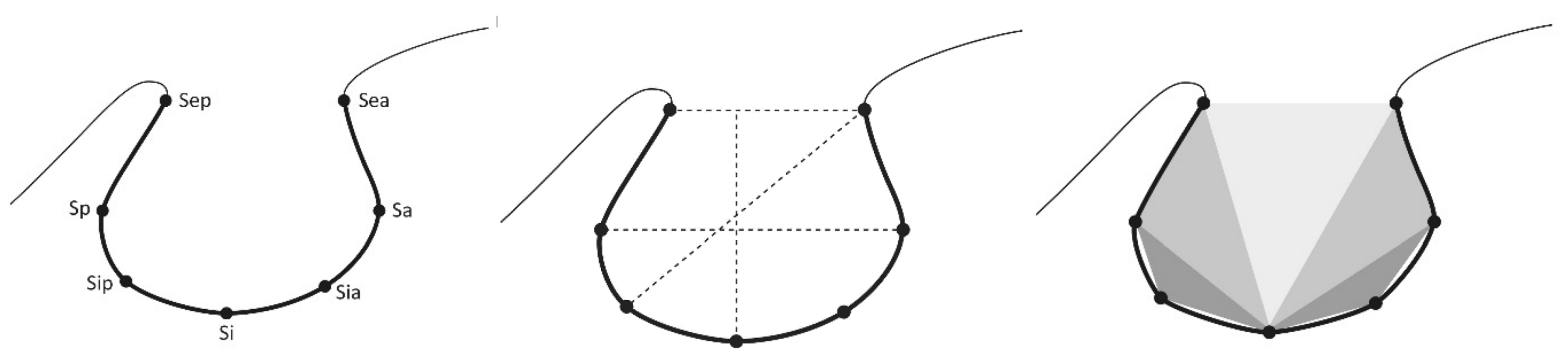
Schematic drawings illustrating the projection of sella turcica on lateral cephalogram: indication of measuring points (left), definition of distances (middle), and sketching of triangulation to calculate sella turcica area (right). Measuring points are defined in Table 5.

The cephalometric procedure did not change during the recruitment time and is described elsewhere in detail [53], [54]. Standardised radiographs were obtained, stored and proceeded as already described [54], [55]. All radiographs showed the region of interest. The radiographs were archived as films. For the purpose of electronic cephalometric analysis, the radiographs were scanned and digitised [55]. A transparent 5x5 cm² film with a millimetre scale was mounted on the X-ray image before scanning. Radiographs were scanned with Agfa Duoscan T1200 using Agfa Fotolook 3.60.00 software (Agfa-Gevaert, Mortsel, Belgium). The software is set to transmitted light registration and a resolution of 300 dpi. Anonymised personal data were registered in Ortho Express^®^ (Computerforum, Elmshorn, Germany). Digitised radiographs were processed with Dental Vision^®^ (Computerforum, Elmshorn, Germany). All radiographs were scanned and processed in Dental Vision^®^ software (Computerforum). Dental Vision^®^ realises the processing and analysis of radiographs from different sources [55]. This software was adapted to the demands of the study and enabled the setting of defined test points of the sella turcica (Figure 2 (Fig. 2)). Measurement points were set on the osseous border facing the cavity. The individually assigned and digitised data were merged into a database which is controlled via a graphical user interface. Images were assessed by two of the authors with experience in X-ray image analysis for more than 25 years and 30 years, respectively. If the definition of a reference point was not unique, the placement of the measurement point was established after mutual agreement. 

### Measurement points

The selection of the measuring points took into account their applicability to cover the area of interest independent of different sella shapes. In cases of double-contour of sella floor the more cranial part was chosen for measurement. When setting the measurement points at the sella entrance (Sea, Sep) the most cranial and posterior/anterior structure equivalent to bone was chosen in order to determine the entrance most accurately. The software allowed calculation of 5 values derived from these measurement points: sella entrance, sella width, sella depths, diagonal of sella and total area (Table 5 (Tab. 5)). Calculation of sella was based on area measurement of five triangulations defined by the measurement points that were added to the total area of this structure. The measurement points and the geometric basis of sella area calculation are illustrated in Figure 2 (Fig. 2). Evaluation of scanned X-rays on a diagnostic monitor and all measurement procedures were performed on a 24-inch monitor in a darkened room.

Prior to the start of the study, the investigation was approved by the local institutional board of the university hospital to fulfil the scientific prerequisite for the preparation of a dissertation in dentistry (J.B.).

### Statistics

We calculated arithmetic mean values and standard deviation of the means. The unpaired t-test was used to compare unrelated samples. Paired t-test was applied to calculate differences between connected measured values. Univariate analysis of variance (ANOVA) and analysis of co-variance (ANCOVA) was performed to study the impact of age and gender on the measurement values. Level of significance for differences measured in this study was set at p<0.05. 

### Quality control of measurements on cephalograms

A second measurement of sella turcica parameters was performed on 30 randomly selected cephalograms three months after first investigation. Differences in measurement values were registered and measurement errors were calculated according to Dahlberg [56] and Houston [57]. Error standard deviation was 0.408818 (Houston) and 0.437226 (Dahlberg). Reliability coefficient of measurements at both time points was 0.993748 and 0.994300, resp. (Houston). 

## Results

### All individuals

**Figure 3:**
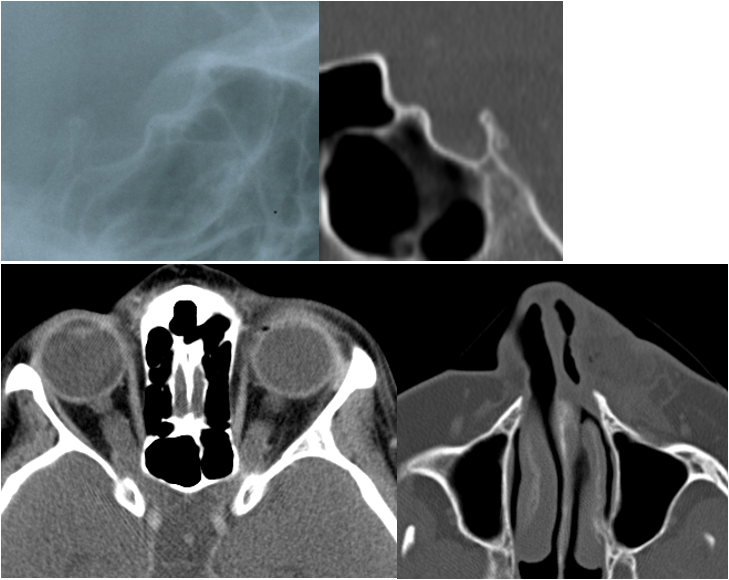
Radiographs of a NF1-affected individual with both OPG and PNF. Upper left side: Cropped image of lateral cephalogram of a NF1-affected 18 year-old female with bilateral OPG and j-shaped sella on lateral radiograph (anterior to the right). Upper right side: Computed tomography image illustrates sella turcica morphology: J-shaped sella on CT (anterior to the left). Sella area is in the range of normal values. Lower left side: Axial CT reveals bilateral enlarged optic nerves. Lower right side: This patient has a plexiform neurofibroma of the left nasal wing and adjacent cheek region.

**Figure 4:**
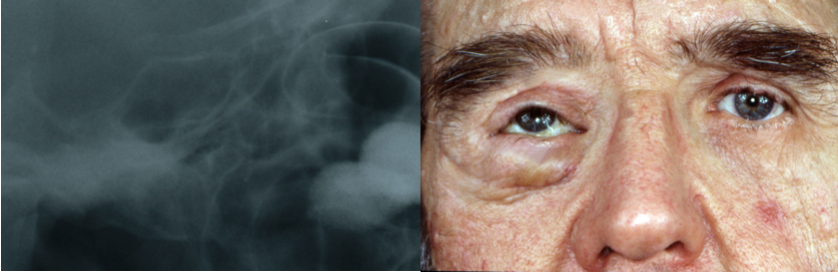
Left side: Cropped image of lateral cephalogram in a 63-year-old male patient with hemifacial PNF: enlarged sella turcica (image of left side). Note eye prostheses and orbital floor augmentation to adjust the prosthesis position to the position of the eye of the unaffected left side. Patient had experienced numerous surgical procedures in the right orbit-temporal region since adolescence. Right side: En-face view of patient wearing eye prosthesis. Note orbital tumour of right side (PNF), vertical difference between eye prosthesis and eye, and deeper position of the right part of nose. The patient also has cutaneous neurofibroma, in particular of the left upper eyelid and front.

The first analysis studied the mean values of all parameters obtained from sella turcica irrespective of gender and age (N=194). These comparisons revealed statistically highly significant differences for almost all measurement values of one PNF subgroup to all other PNF groups and also DCNF and control groups (excepting diagonal of sella). This group constitutes all patients affected with a PNF of the first and second or all three branches of trigeminal nerve. Interestingly, the measurement values of this group were even statistically differentiated from the group of patients who were affected only with one branch of the trigeminal nerve (1^st^ or 2^nd^). The measured values of this unusual subgroup are larger than the values of the others. Furthermore, patients of the DCNF group showed no difference to the other two main subgroups of PNF patients and the control group. Indeed, both DCNF and the control group showed no statistically different mean values of any parameter compared to each other and the two other PNF groups. Double contour of sella floor occurred in 10 cases, predominantly in patients with facial PNF. The results are shown in Table 6 (Tab. 6), Table 7 (Tab. 7), Table 8 (Tab. 8), Table 9 (Tab. 9), and Table 10 (Tab. 10) and illustrated in Figure 3 (Fig. 3) and Figure 4 (Fig. 4).

### Individuals over 18 years of age

The second analysis studied the same parameters in those individuals who were 18 years of age or more (N=148). This calculation takes account of the fact that radiological examinations of lateral cranial projections have proved the growth of this structure in childhood and adolescence. In addition, this restriction takes into account the age distribution of the control group. Analysis was restricted to three main groups (PNF, DCNF, controls). All measurement values of the PNF group were statistically significantly different from both other groups. Furthermore, the study showed that there is no statistically significant difference between the control group and the DCNF group for any of the measured values. This lack of differences allowed the DCNF and control groups to be combined into one group. This group was used for the next step of calculations. The results are shown in Table 11 (Tab. 11), Table 12 (Tab. 12), Table 13 (Tab. 13), Table 14 (Tab. 14), and Table 15 (Tab. 15).

After this impressive demonstration of the dependence of the sella turcica dysplasia on the topography of the facial PNF, a further analysis was carried out in individuals aged over 18 years of age (N=142): patients with facial PNF of the first, second, first and second and of all three trigeminal nerve branch(es) constituted the group ‘upper facial region’. The second group included PNF patients who are affected in the third or second and third trigeminal nerve branch(es) entitled ‘lower facial region’. The comparisons of sella turcica areas proved the area of upper facial region group to be statistically significantly different to all other groups: the sella turcica area is much larger in this group. On the other hand, lower facial region group sella areas did not differ from the control group and DCNF group mean values. The results are summarised in Table 16 (Tab. 16).

#### Calculation of interactions

This analysis focused on the question of whether age and gender had an influence on the logarithmic measured values with respect to study groups. Threshold for age was 18 years of life (Table 17 (Tab. 17)). The study revealed no statistically significant differences in multiple interactions with respect to main groups. Age had the same effect on all groups. Age had a statistically significant impact on sella area in individuals younger than 18 years only (whole study group). Furthermore, in individuals younger than 18 years, sella area was different with respect to gender. However, age and gender had no effect on sella area in patients with PNF. In other words and as an illustration of relevant finding, both a boy of six years and a woman of 77 years showed a similar type of deformed sella turcica with respect to the same type of affected trigeminal nerve. The results are shown in Table 17 (Tab. 17), Table 18 (Tab. 18) and Table 19 (Tab. 19). 

The calculations revealed:

1. Equal impact of age on area in all three groups.
2. Equal impact of age on area in both genders.
3. Equal impact of gender on area in all three groups.
4. Impact of age differs with respect to age group (older or younger than 18 years).
5. Finally, only the factors ‘age’, ‘main group’ and ‘age group >18 years’ show a significant impact on sella turcica area (Table 18 (Tab. 18)).

Significance of factor ‘Under_Over_18*Age’ shows that the impact of age on area is not equally distributed in both age groups. In other words, the impact of age on sella area is dependent on age under or over 18 years. Impact of age on sella area is significant in individuals younger than 18 years of age only. The increase of sella area in this age group is 3.5% per year (regression coefficient 0.015). Pairwise comparison of diagnostic groups reveals statistically significant differences of area measurement values when comparing the PNF group with both other groups. The sella turcica area of patients with DCNF does not differ from sella areas of the control group (Table 19 (Tab. 19)). Differences in mean values show that:

1. The area of the PNF group is 29.72% larger than the area of the control group
2. The area of the PNF group is 30.92% larger than the area of the DCNF group.

**Figure 5:**
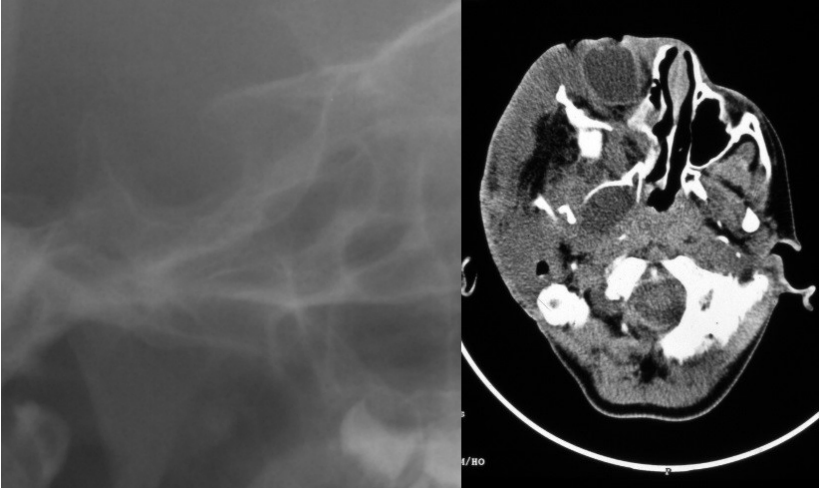
Left side: Cropped image of lateral cephalogram in a 9-year-old male patient with hemifacial PNF: enlarged sella turcica. Right side: Axial section image of computed tomography shows extremely wide foramen ovale on the side of facial PNF.

**Figure 6:**
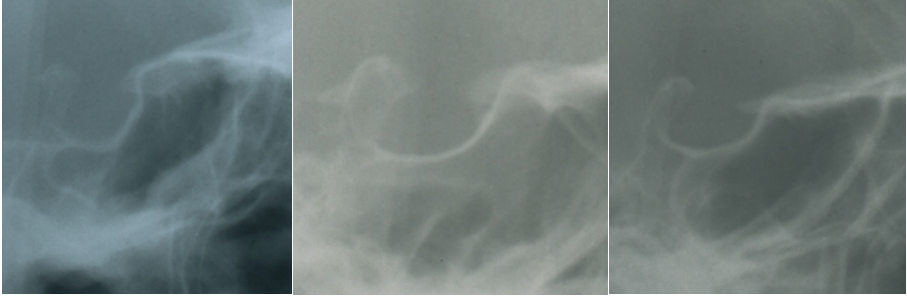
Left side: cropped image of lateral cephalogram of an adult NF1 patient without facial plexiform neurofibroma. Middle position and right side: sella turcica on lateral cephalograms of two individuals of the control group.

Details of lateral radiographs of NF1 patients and control group illustrating sella turcica morphology are presented in Figure 3 (Fig. 3), Figure 4 (Fig. 4), Figure 5 (Fig. 5) and Figure 6 (Fig. 6).

## Discussion

 In this study, the quantified area of sella turcica in patients with NF1 was significantly larger in those who were affected by a trigeminal PNF compared to NF1 patients without facial involvement by a PNF. This enlargement of the sella turcica in trigeminal PNF always occurred in a unilateral facial manifestation of this tumour. The facial PNF showed neither a predilection for gender nor side. With respect to the unilaterally affected trigeminal nerve branches, sella turcica area was consistently largest in size in those patients who showed a tumour affecting one side of the face in all trigeminal branches or tumour expansion restricted to the orbito-temporal region (first and second trigeminal nerve branch). Furthermore, enlarged sella turcica in patients with trigeminal PNF was already detectable in children, in particular in those of the aforementioned group. On the other hand, patients with NF1 and no facial PNF showed a mean size of sella turcica areas not different to the size of sella turcica area in controls. The differences in measurement values with respect to the distribution pattern of facial PNF also apply for the measured distances. It is generally believed that plexiform neurofibromas are connatal tumours [58]. These results put the conclusion close that trigeminal PNF can exert a local effect on the development and ossification of the sphenoid bone during the preliminary stages of sphenoid bone development [59]. However, PNF may also show substantial growth in postnatal life and this growth of a tumorous nerve could also take place at the site of the nerve’s origin [33]. It is reasonable to add the further assumption that this peculiar skeletal malformation of the skull base is only part of a larger interaction field between brain/tumorous trigeminal nerve and bone in this location, giving rise to skeletal deformities, one of them being diagnostic in NF1, and also associated local developmental disorders of brain architecture [60], [61]. The presented results clarify early observations on a correlation between the increased sella turcica and facial PNF [33], [35] by the detection of regularly expected distribution patterns of this facial tumour in the case of the sella turcica deformation.

### Limitations of study

The results of this study have significant limitations concerning their generalisation on the NF1 phenotype.

1. Although standardised radiographic skull projection was maintained, errors of measurement are inherent to cephalometric analysis [57]. These measurement errors are of particular importance when attempting to detect small quantitative differences in a parameter [56]. However, we achieved substantial agreement of the measured values at different examination times. Nevertheless, the determination of bone limits was demanding.
2. The analysis is restricted to the radiological appearance of sella turcica on a standard lateral radiographic projection. The main content of sella turcica is the pituitary gland. No assumptions on associated pituitary morphology [62], [63], [64], [65] structures adjacent to sella turcica [65], [66], and pituitary functions [41] can be drawn from this analysis. Nevertheless, development of the pituitary and sella turcica are closely related [39]. Based on animal studies, the gene product of *NF1*, neurofibromin, is involved in the regulation of body growth via the hypothalamic-pituitary axis [67]. In addition, this analysis cannot take into account further individual parameters such as body height for comparison with sella turcica area measurement values.
3. The restriction of analysis to an osseous compartment of skull base cannot clarify the impact of adjacent tissues on sella turcica morphology. Indeed, it has been emphasised that quality and information obtained with modern cross-sectional imaging techniques of the sella region have largely rendered superfluous plain X-ray investigations [68], [69].

Possible local influencing variables of importance in assessing the sella turcica on lateral radiographs include:

1. OPG with extension to optic chiasm [37], [70], [71],
2. vascular anomalies, in particular of internal carotid [72],
3. neoplasms of the pituitary gland associated with NF1 [45], and
4. connatal malformations of the brain in close proximity to the middle cranial fossa.

However, these findings are extremely rarely published in the context of NF1, excepting OPG [71] and brain dysmorphology associated with sphenoid bone dysplasia. In addition, it should be kept in mind that it was repeatedly reported to have revealed primary osseous malformations of the skull base without evidence for adjacent plexiform neurofibroma [23] and it is common sense that skull defects in NF1 do not necessarily correspond to the total area of an adjacent PNF [19], [73]. Finally, a recent cephalometric study stressed the importance of a putative haploinsufficient bone of the skull base to explain distinct cephalometric findings of this region [46].

Ad 1. OPG are diagnosed in about 15% of patients with NF1 (for review: [71]). NF1 associated OPG preferentially affect the anterior visual pathway, i.e. intraorbital and chiasmatic parts [71]. Coincidence of OPG and facial PNF was occasionally noted in an earlier report on OPG in NF1 [74], but this claimed pathogenetically related association of findings was based on a small sample size of affected patients and could not be substantiated in a recent study on OPG in NF1 [71]. Indeed, there is no overlap between this recent study group of OPG in NF1 [71] and the present study group of lateral cephalograms of patients with NF1. In addition, detailed radiological studies on dysmorphology of the sphenoid have repeatedly shown the close association of PNF and the distorted bone, but never mentioned the coincidence of OPG and PNF [22], [36], [75], [76] and appears to be rare [71]. However, OPG can exert a well-recognised skeletal modelling on the anterior skull base. Here, sometimes a flattening of the chiasmatic groove (syn. prechiasmatic sulcus or *sulcus fasciculi optici*) is seen on lateral X-rays of the skull [77], [78]. This phenomenon is known as a radiological metaphor under the term “j-shaped sella” [68], [78] but is not pathognomonic for OPG [79]. Furthermore, it was already clear from evaluation of plain radiographs in neurofibromatosis patients that j-shaped sella may occur without an association with any optic lesion [35], [80], [81], [82] and appears to be rare in NF1 [36]. Investigations of OPG in children based on plain radiographs lead to the conclusion that the sphenoid very likely accommodates itself to a growing mass within the optic chiasm or nerve and thus the tumour may not be visible as an imprint on bone. This assumption was used to explain the lack of j-sella in patients with tumours starting later in life [78], [81]. Furthermore, enlargement of the optic nerve as a consequence of an OPG does not necessarily deform the chiasmatic sulcus [83]. The sulcus does not contain the chiasma [65]. On the other hand, optic foramina may be normal in the presence of a so-called j-shaped sella [84]. Prior to the invention of computed tomography, this finding was repeatedly debated to address distinct pathologies of this region, including OPG [78]. However, detailed analyses of this region have shown that misinterpretation of normal sphenoids entice to diagnose a “j-shaped” sella, [85]. Nowadays, this finding is not regarded a first-line radiological finding for the description of cranial-base pathologies. Widespread and fast access to advanced cross-sectional and functional imaging techniques allow detailed multi-dimensional investigation of the region of interest [68], [86]. Indeed, a j-shaped sella is a well-documented, not infrequent finding on lateral cephalograms of healthy children and adolescents [87]. Therefore, this finding was regarded a radiological variant in this age group [78], [87], but association with certain pathologies was repeatedly emphasised, even recently [88], [89], [90], [91]. Indeed, the association of a j-shaped sella and OPG in NF1 is still a teaching content of textbooks of cranial radiology [68] and clinical reviews on OPG [92]. In this study, the frequency of j-shaped sella was 5 cases in NF1 patients (3%, all children and adolescents) and in the range of expected variants of normal sella turcica radiomorphology on plain lateral projections, in particular in children [78], [87], [93]. Out of this group, one NF1 patient with j-shaped sella turcica on lateral cephalagram proved to be affected with bilateral OPG. Surgery had been repeatedly performed for a diffuse PNF of one side of the nose. This exceptional case is presented in Figure 3 (Fig. 3).

Ad 2. NF1 is also a disease of the vascular system [5]: With respect to the region of interest of this study, however, intracranial carotid artery dysplasia would likely be the most important vessel that could exert a pressure effect on adjacent bone [94]. Intracranial aneurysm is a very rare phenomenon in NF1 [72], and this assessment also applies to other lesions of the intracranial carotid artery in this disease [95], [96]. We performed no angiography to exclude vascular anomalies of the region of interest [79]. However, a statistically significant effect of unrecognised aneurysms of this region on the sella turcica which is effective to alter sella turcica shape and area is very unlikely.

Ad 3. Besides a general effect of the central nervous system on bone (short stature, early onset of osteoporosis) in NF1 [67], special abnormalities of pituitary function are occasionally noted in patients with NF1 [97]. In many cases, these endocrinologically active malfunctions of the pituitary are caused by the pressure and invasive growth properties of an OPG [98]. A rapid mass effect of an OPG is likely to be effective much earlier on soft tissues that are not capable of evading bony environments than on the surrounding bony capsule itself. However, this assumption could not be proven in this study: neither magnetic resonance images nor endocrinological investigations were performed in this patient group. Furthermore, this putative effect of OPG on bone could also be age-dependent [81]. OPG develop preferentially in children younger than 7 years of age [71]. In addition, it is to be pointed out by way of example the case for (primarily cystic) intrasellar astrocytomas that could exert internal pressure on the bone as a complement to the pathogenesis of external pressure on the sella region by OPG [44]. The subject assessing the hypothalamic-pituitary functions in NF1 gets even more complicated when taking into account that signs of precocious puberty in NF1, e.g. gynaecomastia, may develop without detectable hormone imbalance leading the investigators to the conclusion endocrine diagnostics appear to be unnecessary in these patients [99]. Furthermore, the putative causal relation between the rare case for primary pituitary adenoma and NF1 is presently unclear [100]. Table 20 (Tab. 20) lists some rare pituitary tumours that could cause sellar deformation [101], [102], [103], [104]. In addition, radiology of sella turcica size only rarely shows an altered bone as a result of primary pathology of the pituitary on incidence [30], [68]. 

Ad 4. Dysplastic brain is frequently seen on the same side as facial PNF on cross-sectional images. Growth effects of adjacent dysplastic brain could also influence the sphenoid bone development. This factor should be analysed in future studies using adequate imaging techniques allowing both soft and hard tissue investigations [60], [61]. 

In summary, it must be noted that this investigation cannot rule out the possibility that (undiagnosed) OPG or further soft tissue pathologies with neoplastic properties other than trigeminal PNF may have had an influence on the shape or area of the sella turcica in patients of this study. However, accurate analysis of the relevant literature make these findings very unlikely as influencing factors on the results of the investigations (Table 20 (Tab. 20)). The effect of dysplastic brain cannot be assessed with this method.

Primary osseous dysplasia of the skull without detectable adjacent (peripheral) nerve sheath tumour is a long-standing and well-known peculiarity of NF1 [1], [23]. However, current assessments of typical NF1-associated cranio-facial dysmorphies assume that PNF is present in direct topographic relation to the lesion [22], [36]. Indeed, the density and amount of PNF inside a tumorously transformed region can vary widely. The extent of skeletal deformation in topographical proximity to a PNF does not necessarily match the assumed tumour extension as revealed on cross sectional images [105]. It is probable the time of tumour formation during the first phases of life that is also relevant for this variance of the dysmorphies as are genuine growth properties of the tumour and bone. A recent study points to the probable effect of haploinsufficient bone in NF1 on the growth of the skull base, in particular the sphenoid [46]. With respect to the measurement values of DCNF patients, this putative effect will not interfere with sella turcica size and shape as depicted on lateral radiographs. 

### Intracranial topography of trigeminal nerve

Sella turcica area is impressively enlarged in patients with hemifacial PNF and this finding stands out against sella turcica areas in patients with trigeminal PNF restricted to one or 2 branches. This difference in size of the area is statistically significant in all comparisons. It can only be assumed that this limitation of the tumour extension is reflected morphologically also in the entire proximal part of the nerve branches. In fact, the proximal parts of the nerve that will divide into branches are already arranged topographically in their origin [65]. With reference to the layered structure of the trigeminal nerve at the skull base, it appears worth studying the quantitative relationship between the intracranial tumour volume and the size and shape of the sella turcica. However, it has to be considered that there is no knowledge of the expected growth of the PNF from the trigeminal ganglion during the embryonic phase and early childhood. Therefore, the suspected skeletal effect of the tumour at the origin of the nerve is difficult to determine in the lifetime.

### Qualitative changes in the sella contour

This study was restricted to measurements of the sella turcica and does not take into account skeletal anomalies of this region such as sella bridges or differences in shape of *dorsum sellae* or *tuberculum sellae* [39]. Restriction of point setting to the upper contour in cases of double floor imaging was at risk to underestimate the area. Error of projection and missing a strictly lateral projection of the X-ray beam onto the object is the most likely cause of seeing a double contour of sella turcica floor on lateral skull radiographs [106]. On the other hand, radiologists know about the double contour of sella floor on lateral radiograph as a result of side-uneven shape of sphenoid sinus’ roof [107]. This differentiation between incorrect positioning of head and true asymmetry of sinus would have been facilitated with the aid of tomograms perpendicular to the X-ray projections under study [105]. This distinction was not feasible in this study due to the study design and the lack of indication to perform routinely computed tomography in these patients. It is not necessary to emphasise that the assumption of a straight surface for the sella floor does not correspond to the anatomical contour of this region [65]. The number of patients with double contour of sella do not differ from data of recent reports on this item [108].

Asymmetries in the vertical dimension of sella turcica can have a serious effect on skull base surgery procedures in NF1 patients. In one case report, no plain radiological radiograph of the skull adequately described the special feature of an asymmetrically formed sella turcica and deformation of the roof of the sphenoid sinus in surgical planning for ventriculostomy in hydrocephalus [105].

### Comparison to other studies

Measurements of sella turcica have been performed for decades both on skeletal remains and on radiographs [108], [109], [110]. However, one of the main results of many studies is the impressive variability in the size and shape of sella turcica [29], [108]. Table 21 (Tab. 21) provides an overview on selected published studies using radiographs to measure sella turcica lines and areas. This comprehensive overview illustrates the strong dependency of the results from the technical parameters of radiography and the difficulties in comparing results of studies that have applied variable parameters and have differed in the selected calculation basis. Therefore, it is reasonable to discuss the results of this study in the context of literature with caution. However, the very large sella turcica areas in cases with pituitary adenoma measured by Krennmair et al. [69] once again make clear the earlier misinterpretation of the increased sella turcica size on lateral radiographs of NF1 patients as a radiological indication of a pituitary tumour [33], [35] (Table 21 (Tab. 21)). Several studies revealed that human sella turcica shows continuing growth with age, in particular in children and adolescents [108], but apparently also in adults [109]. Area measurement values of this study in healthy individuals are similar to those of Israel [109] and Krennmair et al. [69], but higher than those of Andredaki et al. [29]. Mean value of sella entrance of the control group is between those published by Korayem and Alkofide [91] and Axelsson et al. [108], but is higher than in a Greek population study [29]. Sella width is slightly larger in controls of this study compared to mean values of this item provided by Krennmair et al. [69], Andredaki et al. [29], but smaller than in the investigation of DiMario et al. [36]. These comparisons reveal that differences in results are likely to be attributed to differences in the definition of landmarks as well as differences in the populations studied. As a consequence of these differences in the results of the literature, the presented own results are considered to be objectively valid within the study. However, due to the inaccuracy of the measurement inherent in this imaging technique and variations in defining radiological landmarks, a separate calibration of the measurement points and evaluations is necessary for each additional study.

## Conclusion

This study provides evidence for the topographical relationships of the type of facial PNF and sella turcica deformities on lateral skull radiographs. Increased sella turcica area is strongly associated with a definite phenotype of NF1 patients affected with facial PNF. These findings may have some significance for the assessment of associated pathologies in the reconstructive surgery of the face of these patients. Furthermore, these results are valuable for the planning of surgical procedures on the cranial base in patients with NF1. On the basis of the presented findings, it must be assumed that the deformations of sella turcica in the defined patient groups have already manifested in childhood and are likely embryological in origin. The results of the examination are also a contribution to the knowledge about the skeletal dysmorphism of this syndrome.

## Notes

### Competing interests

The authors declare that they have no competing interests.

### Acknowledgement

The authors thank Mrs. A. Rusche, Computerforum, Elmshorn, for adapting the software Dental Vision^®^ to the requirements of this study.

### Authorship

The authors REF and JB contributed equally to this publication.

## Figures and Tables

**Table 1:**
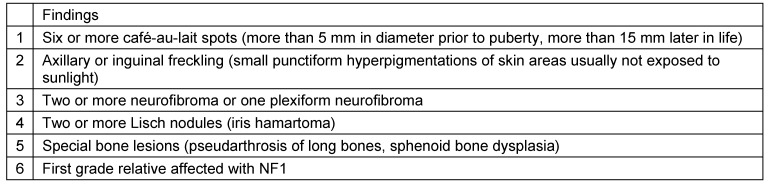
Diagnostic criteria to establish NF1 diagnosis [1]. At least two criteria have to be identified in an individual to diagnose NF1.

**Table 2:**
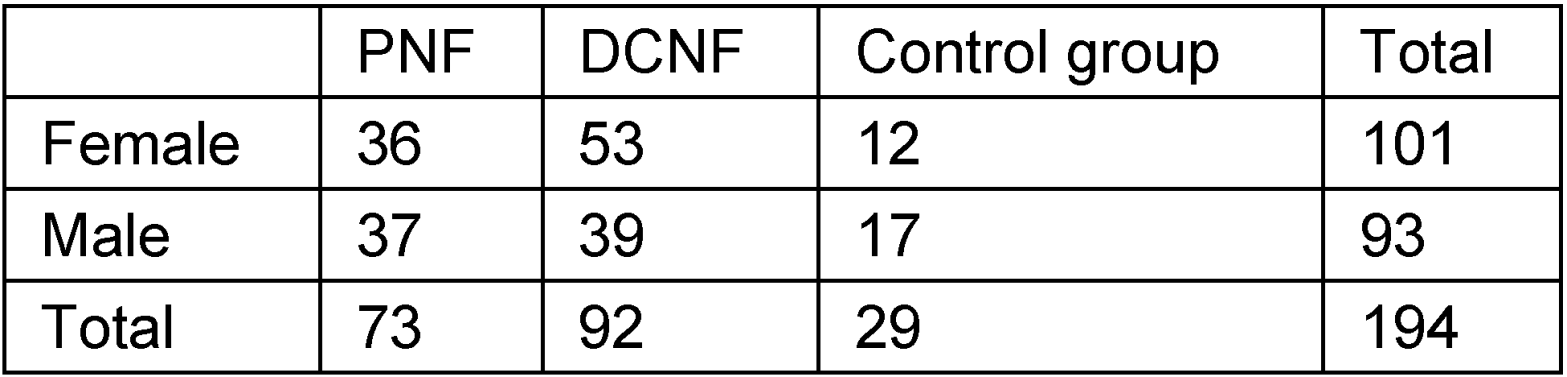
Composition of study groups

**Table 3:**
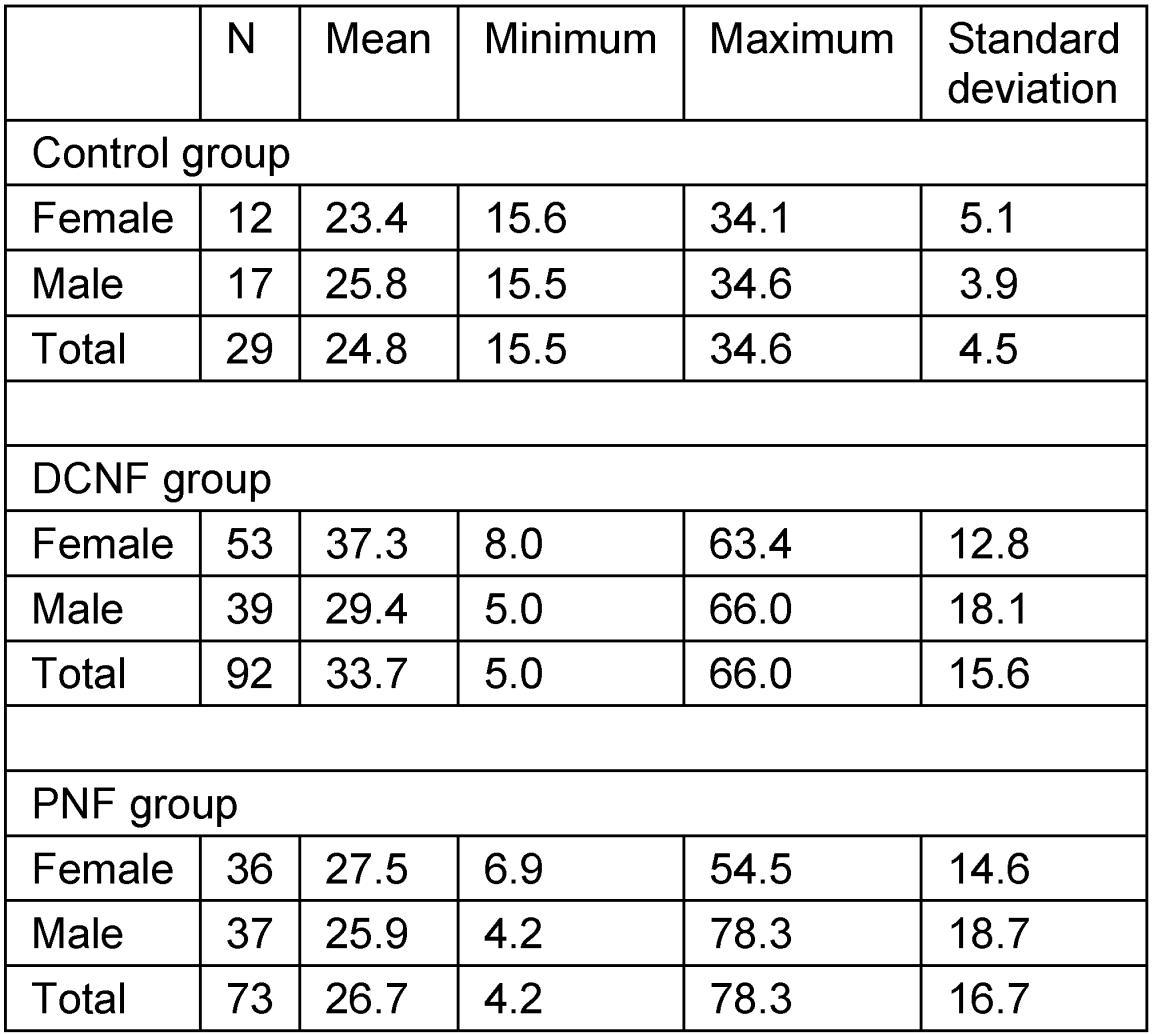
Age distribution of study groups (years)

**Table 4:**
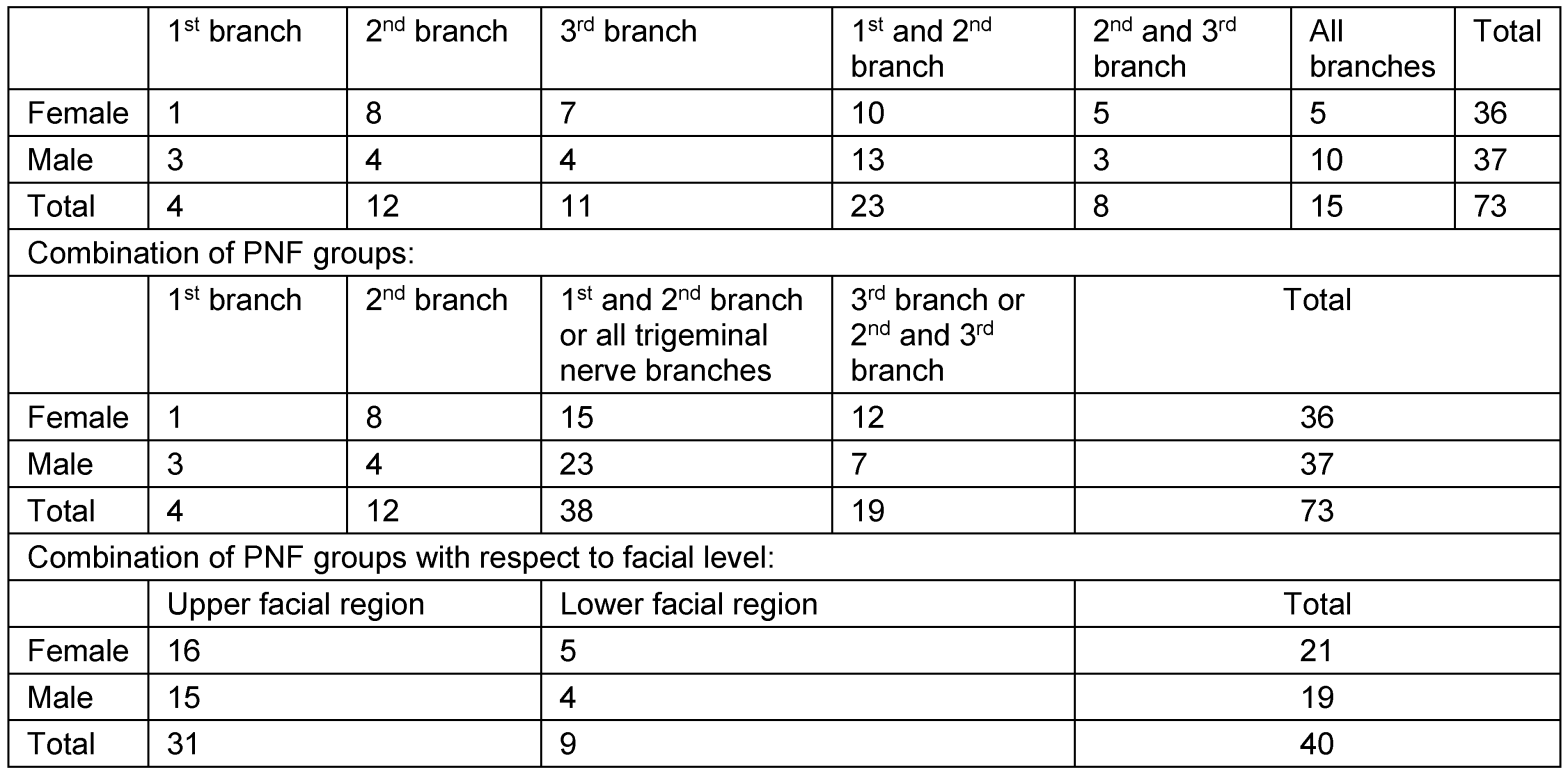
Subdivision of PNF group with respect to affected trigeminal nerve branch(es)

**Table 5:**
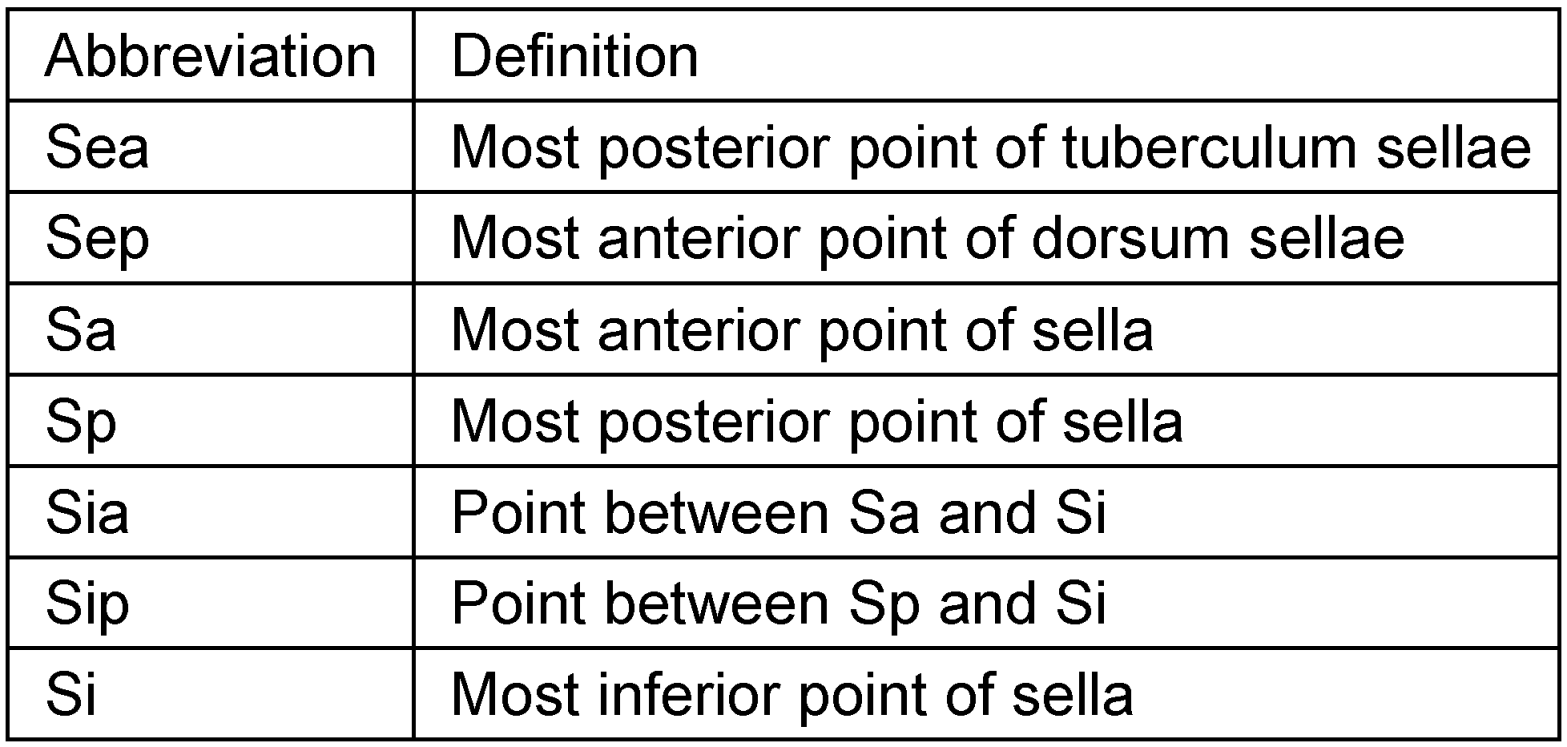
Definition of measurement points of sella turcica on lateral cephalograms

**Table 6:**
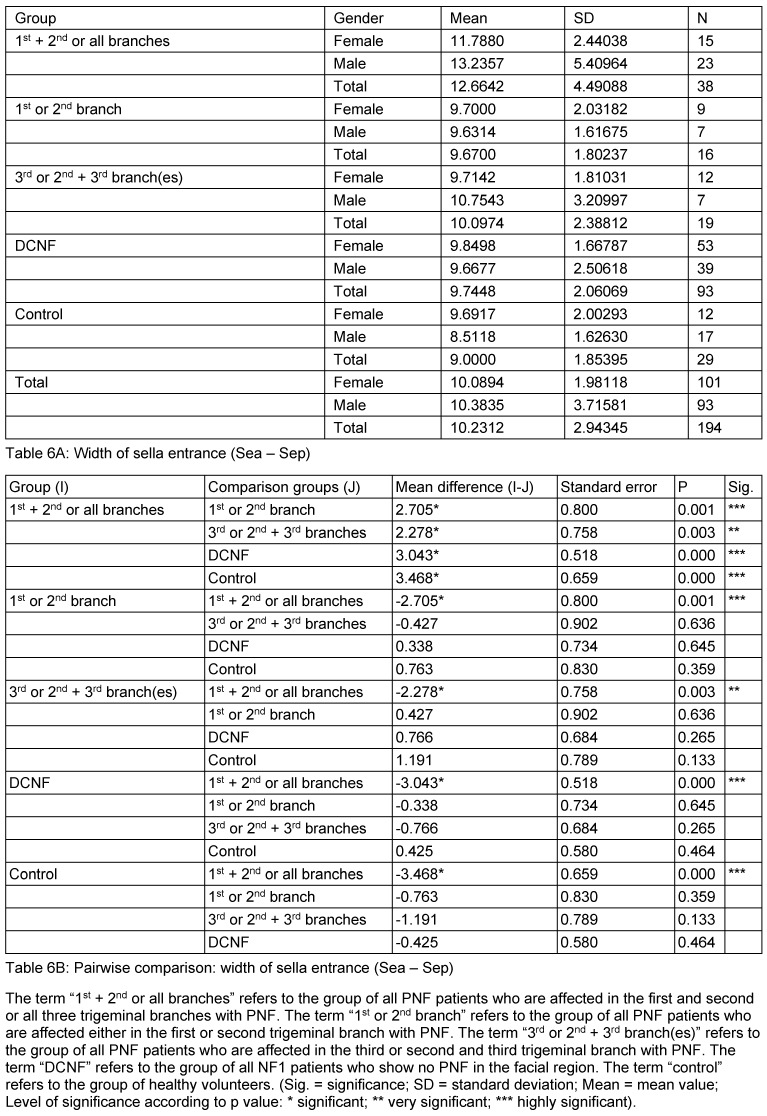
Mean values and pairwise comparison of measurement values detailed for the sella entrance width

**Table 7:**
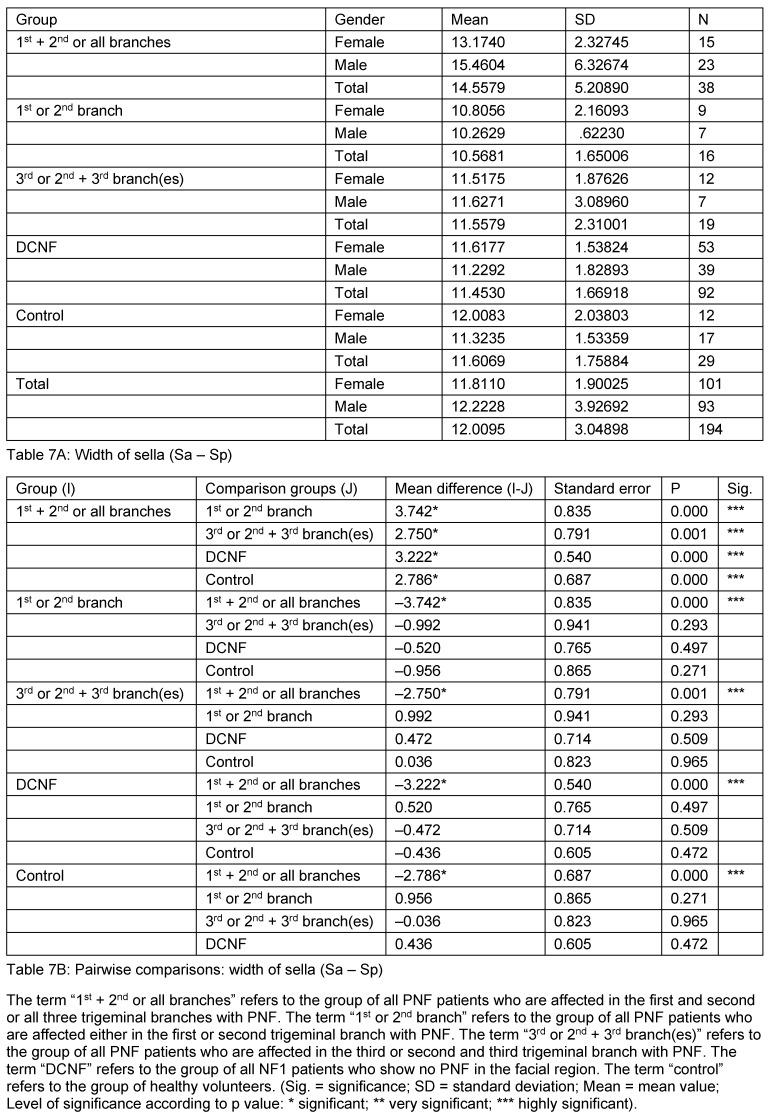
Mean values and pairwise comparison of measurement values detailed for the sella width

**Table 8:**
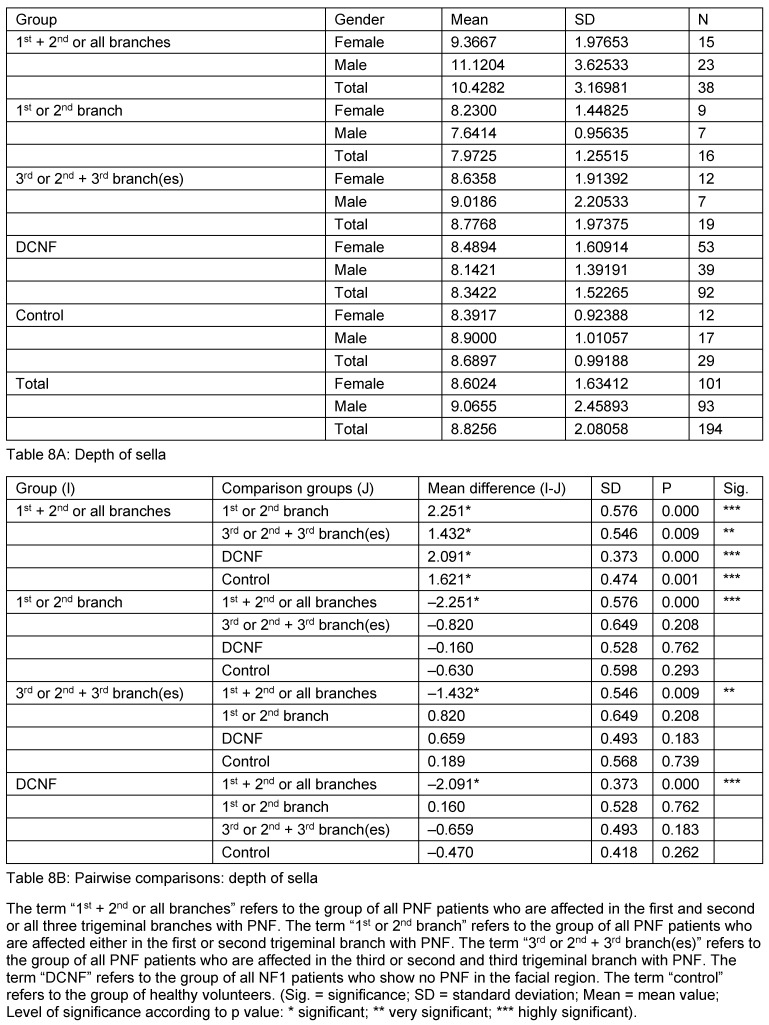
Mean values and pairwise comparison of measurement values detailed for the sella depth

**Table 9:**
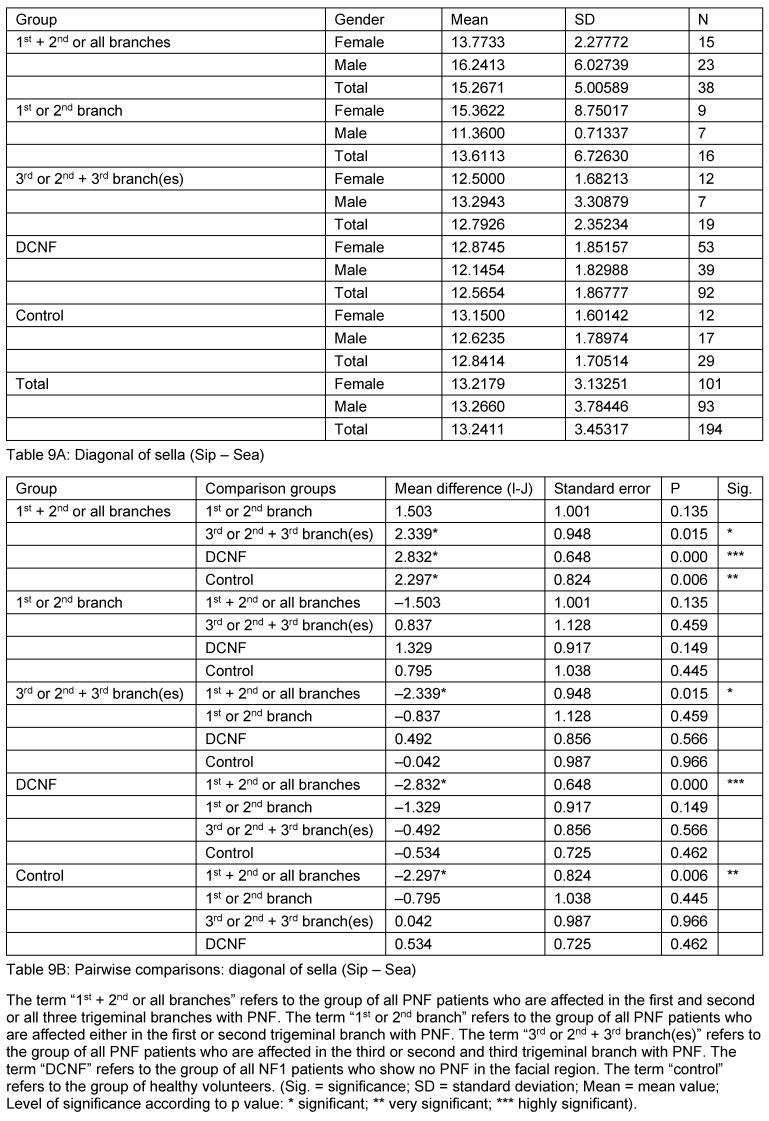
Mean values and pairwise comparison of measurement value detailed for the sella diagonal

**Table 10:**
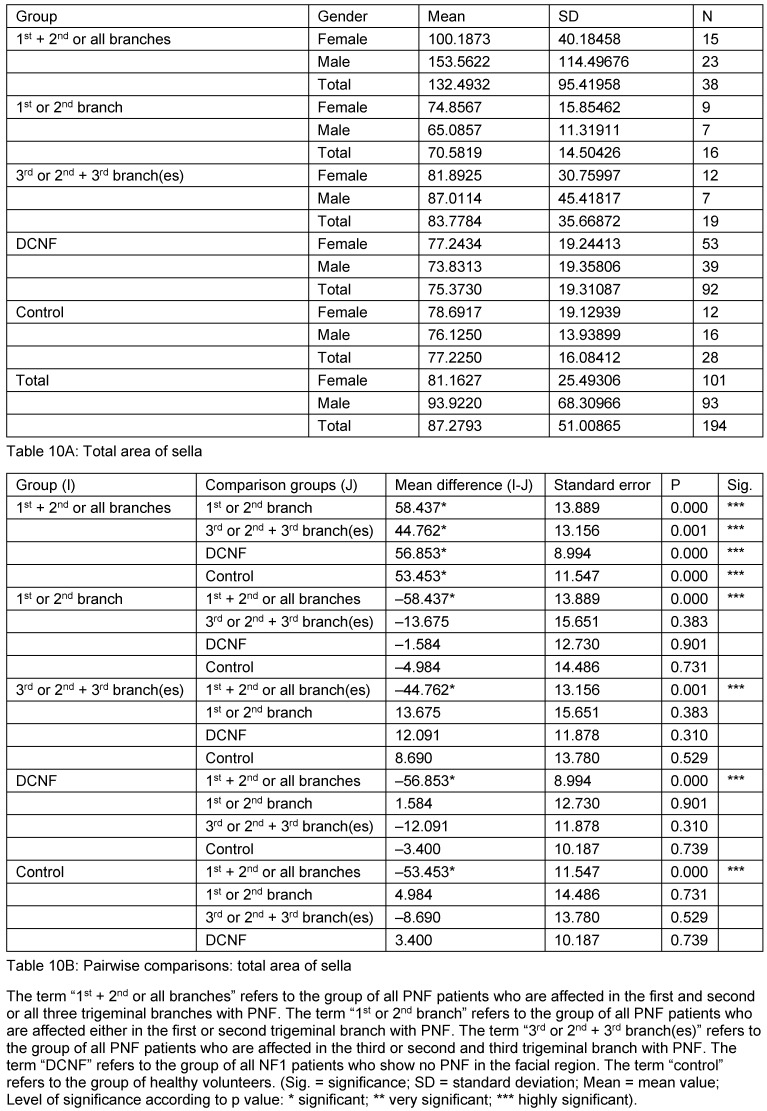
Mean values and pairwise comparison of measurement values detailed for the sella area

**Table 11:**
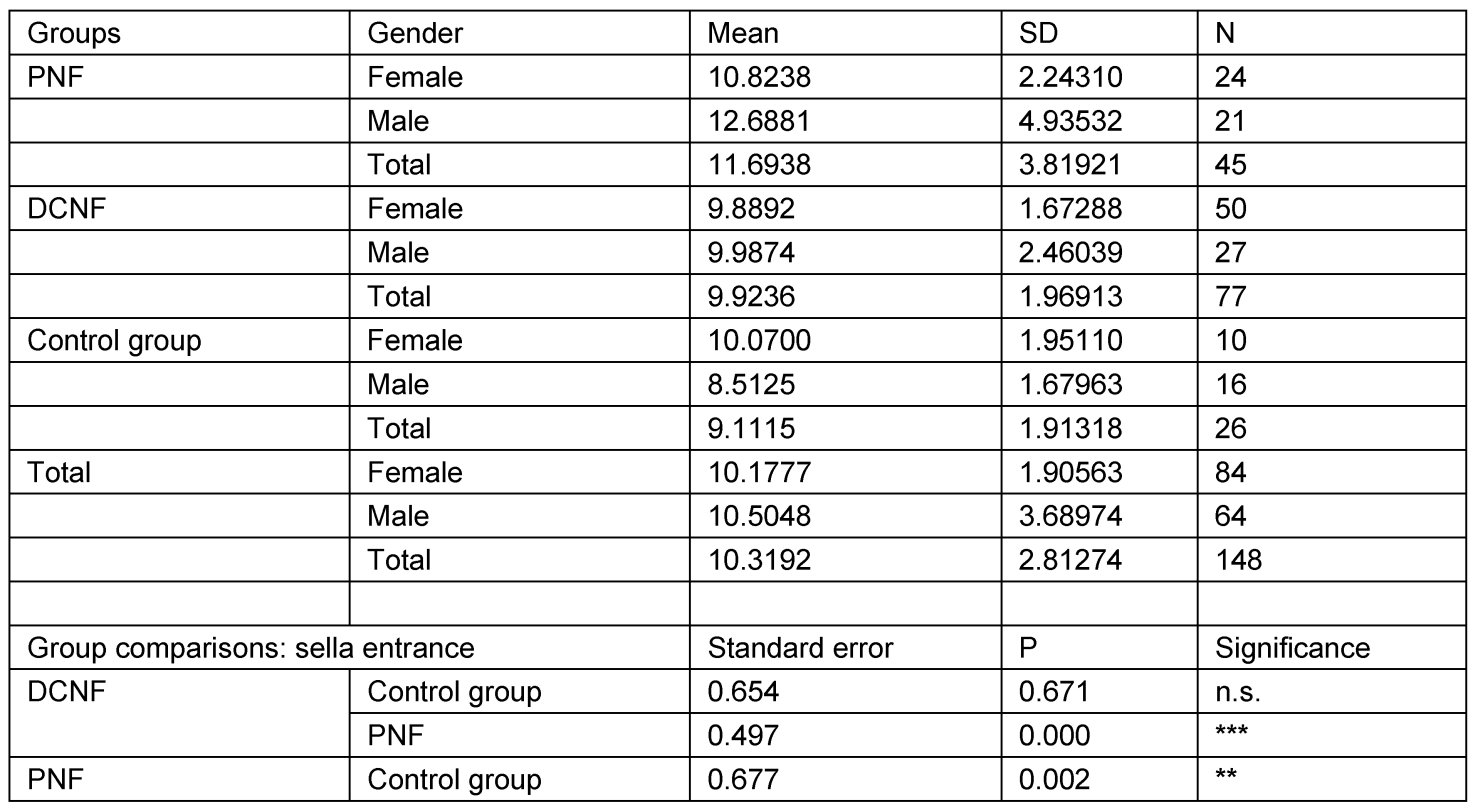
Analysis of sella entrance measurement values in individuals aged over 18 years and pairwise comparison of mean measurement values with respect to diagnostic group

**Table 12:**
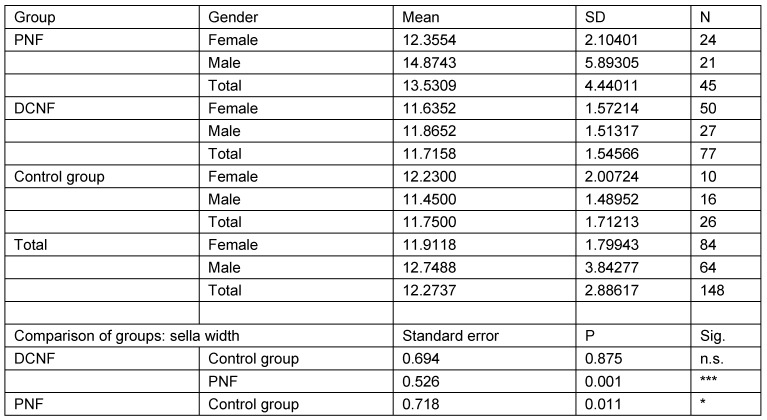
Analysis of sella width measurement values in individuals aged over 18 years and pairwise comparison of mean measurement values with respect to diagnostic group

**Table 13:**
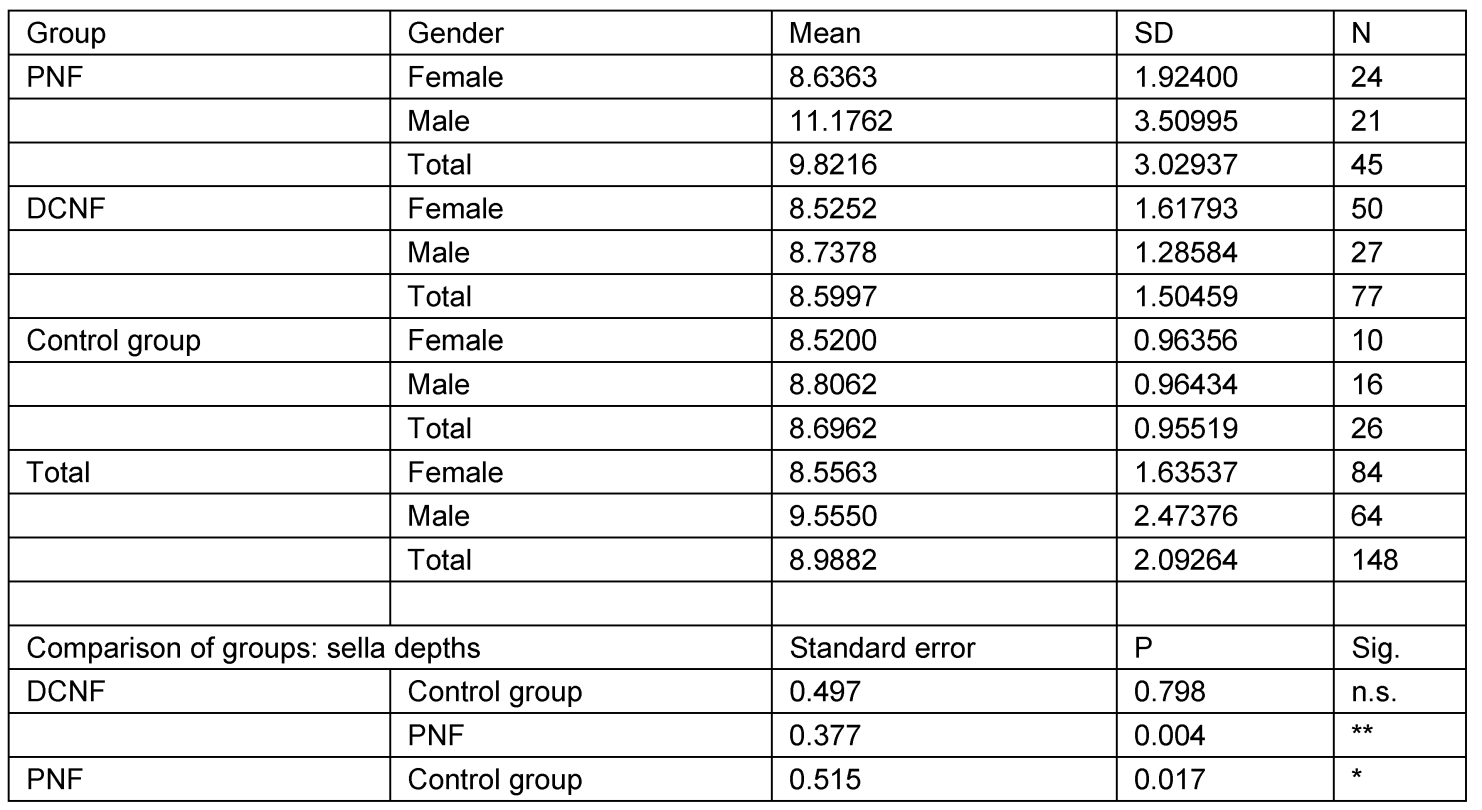
Analysis of sella depths measurement values in individuals aged over 18 years and pairwise comparison of mean measurement values with respect to diagnostic group

**Table 14:**
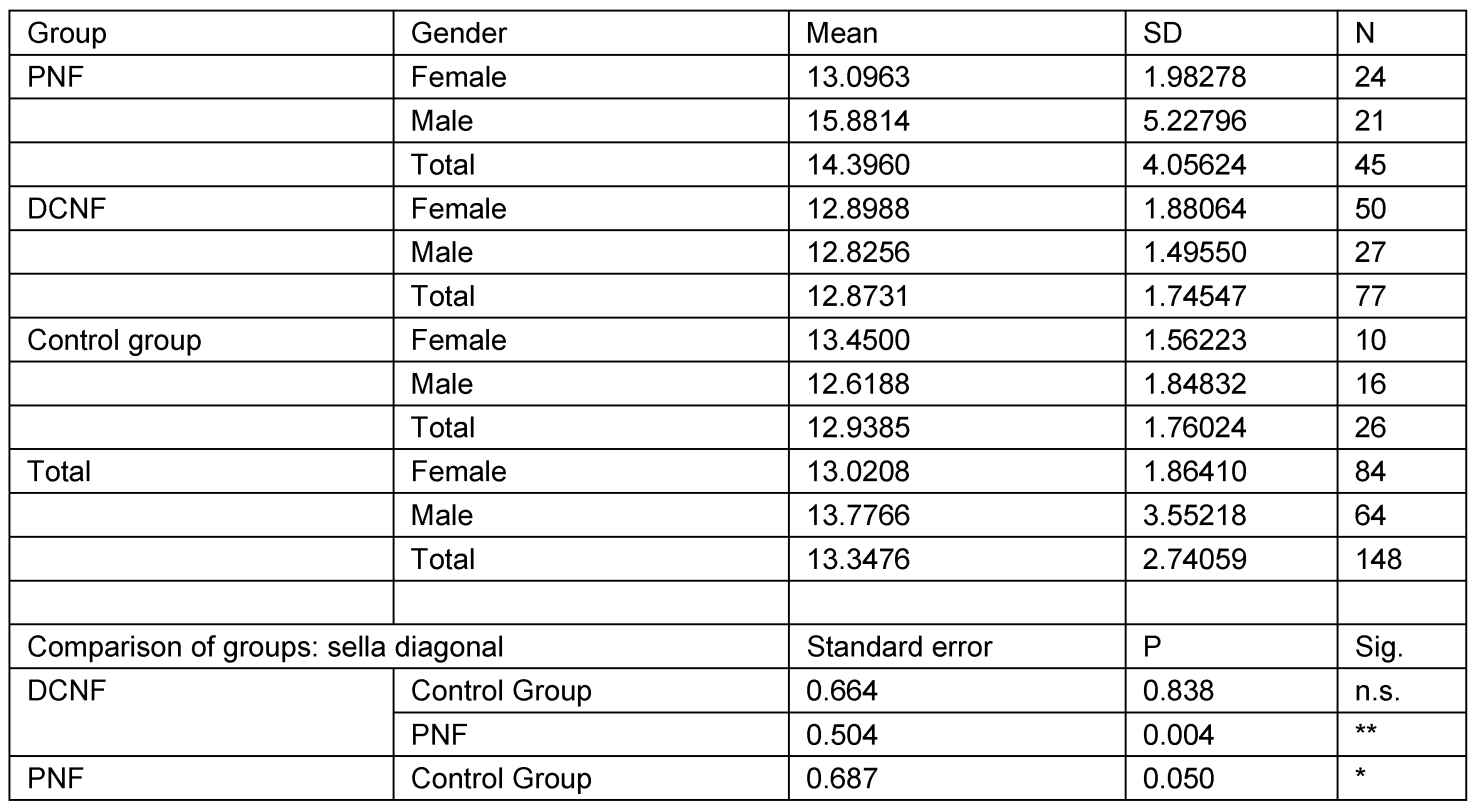
Analysis of sella diagonal measurement values in individuals aged over 18 years and pairwise comparison of mean measurement values with respect to diagnostic group

**Table 15:**
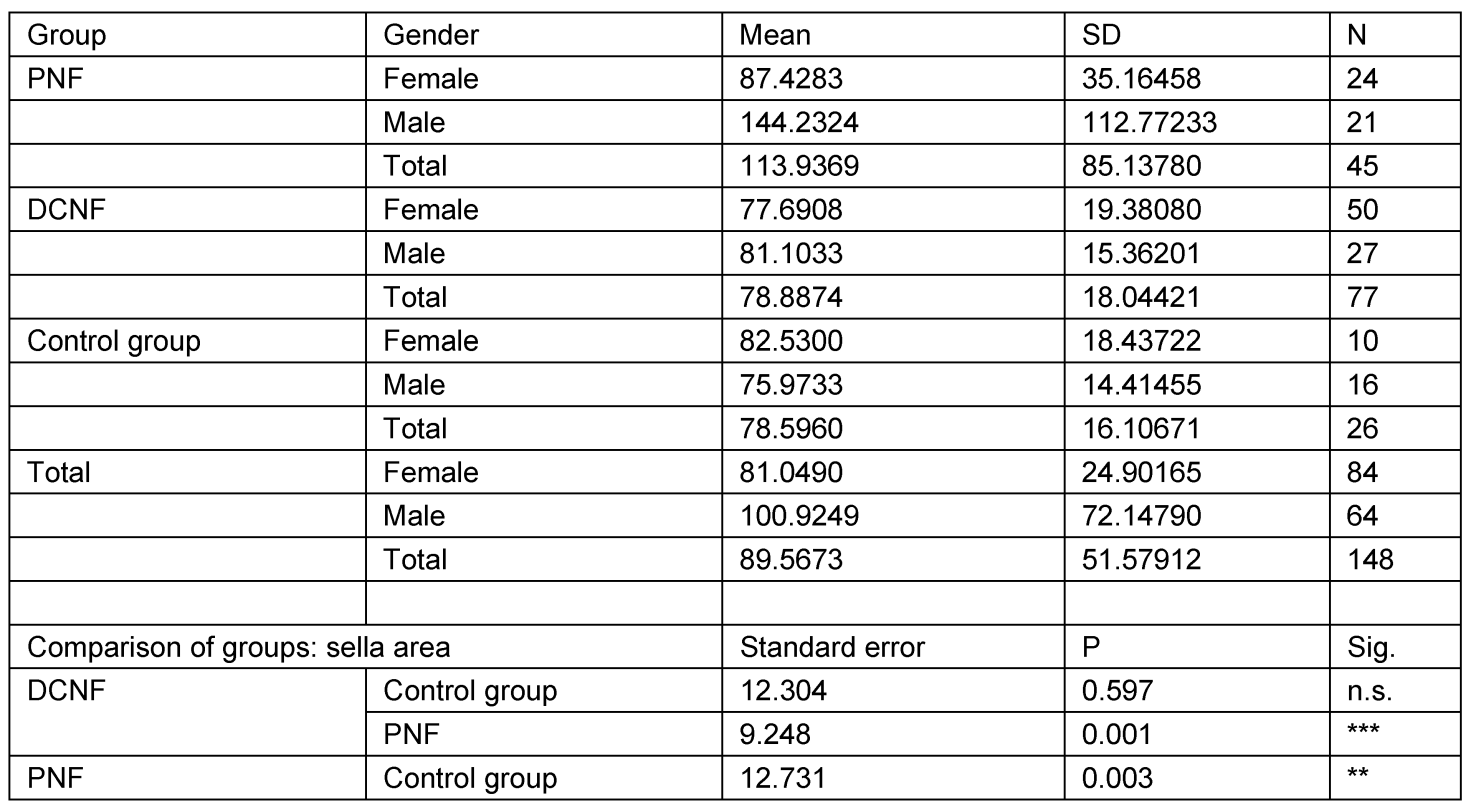
Analysis of sella area measurement values in individuals aged over 18 years and pairwise comparison of mean measurement values with respect to diagnostic group

**Table 16:**
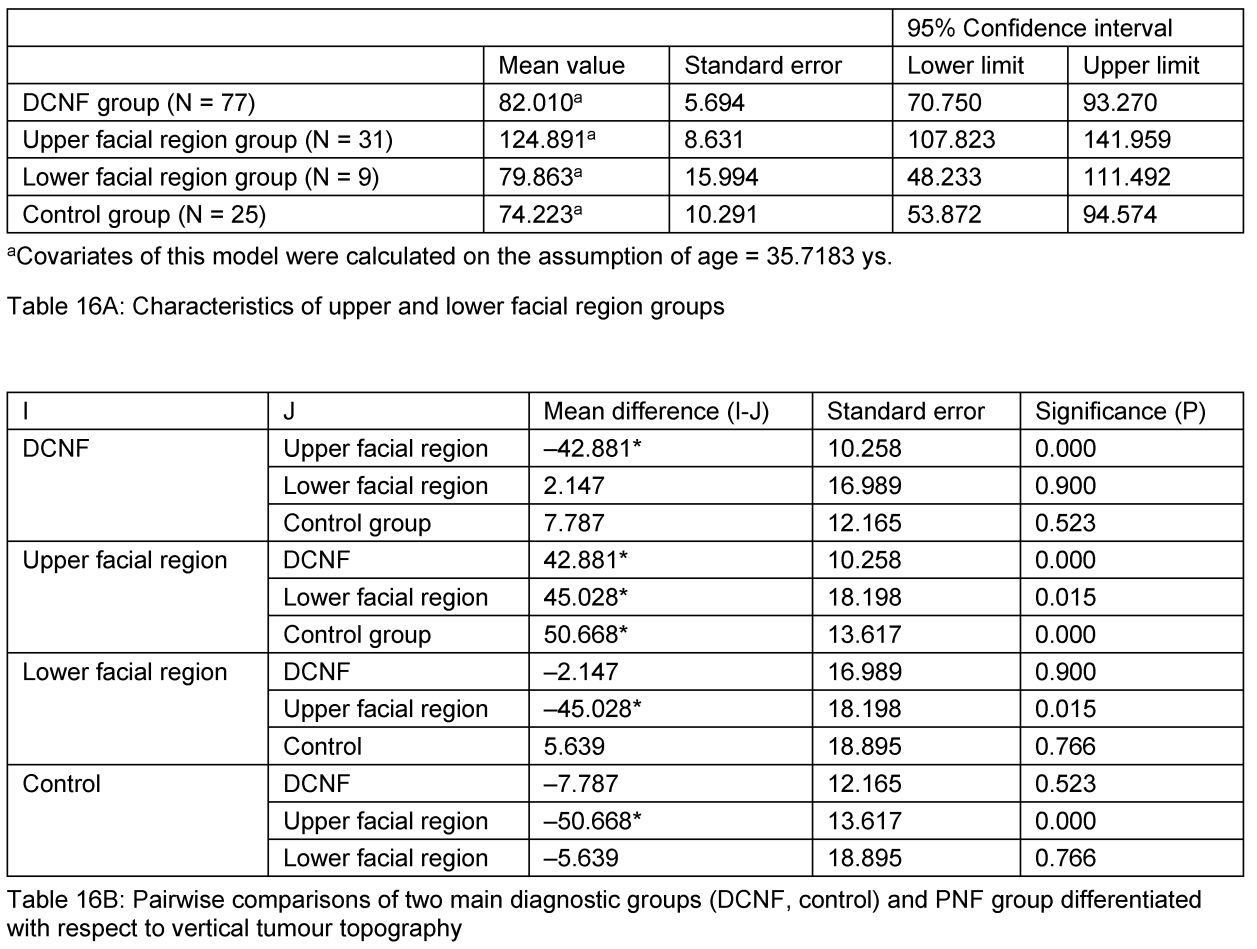
Comparison of sella turcica area in patients with NF1 (PNF or DCNF) and controls. All individuals are aged 18 years or more. PNF group was subdivided in two groups: ‘Upper facial region’ group in the context of this analysis includes all patients who are affected in the first, the second, the first and second or all three trigeminal nerve branches. ‘Lower facial region’ group includes all patients who have developed a PNF confined to the third or second and third trigeminal nerve branch(es). Table (A) describes characteristics of groups and (B) illustrates pairwise comparison of groups.

**Table 17:**
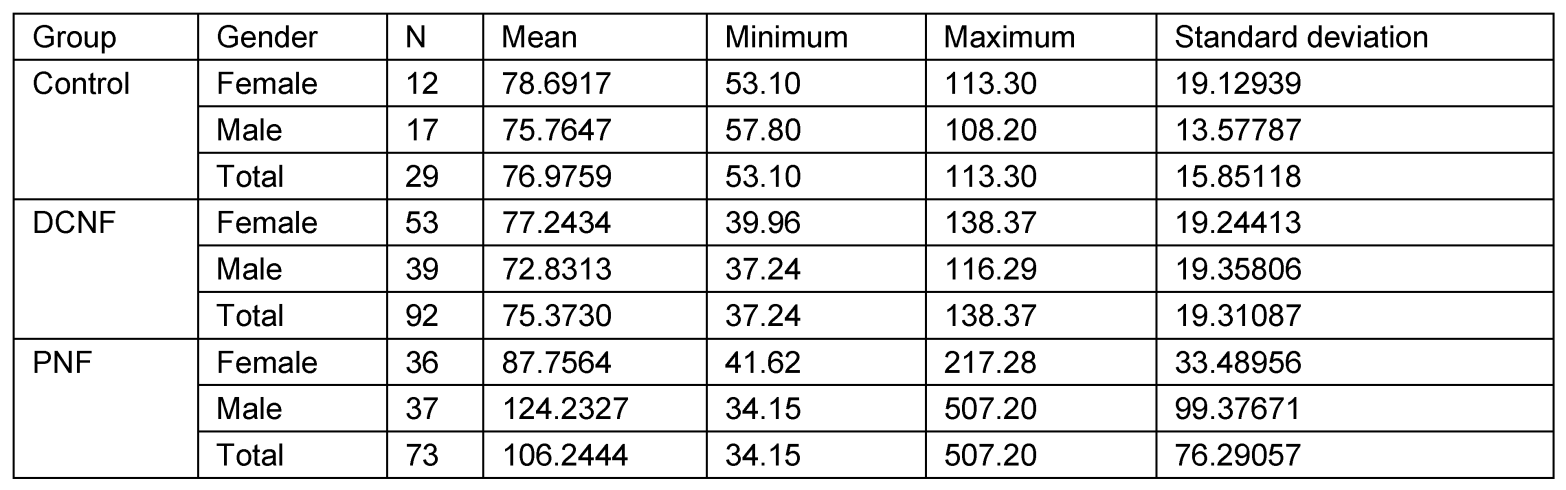
Characteristics of diagnostic groups. Univariate ANOVA of logarithmic total sella turcica areas was used to compare the three main diagnostic groups (control, DCNF, PNF) with respect to age (younger or older than 18 years of age) and gender

**Table 18:**
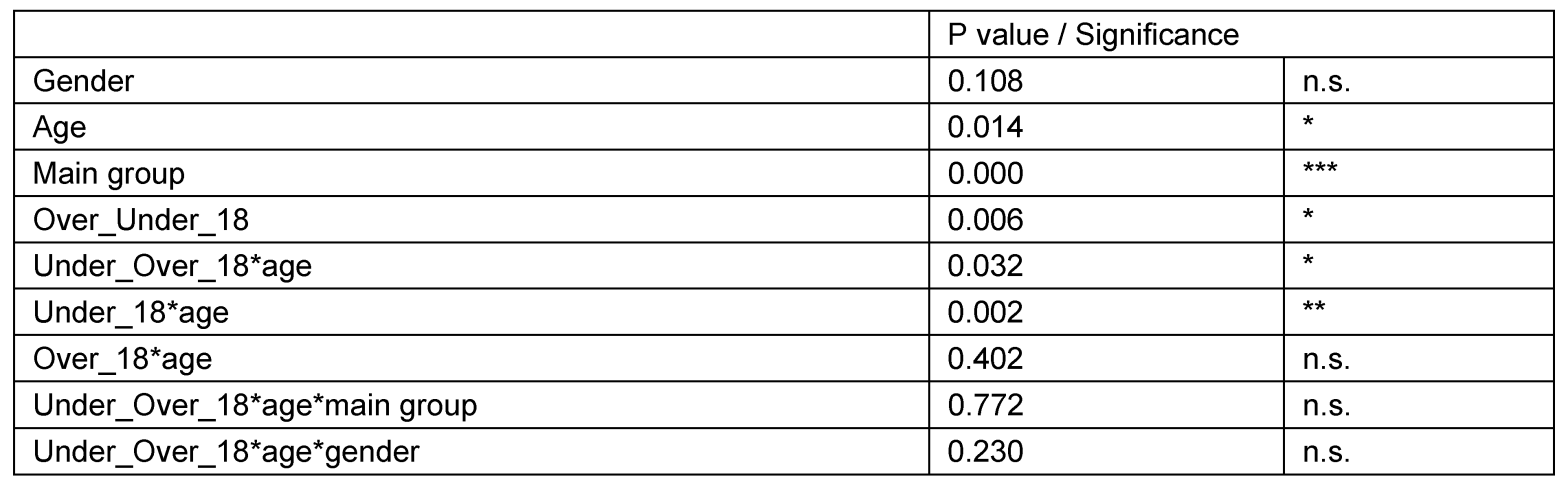
Impact of factors on sella turcica area following calculations of double or threefold interactions

**Table 19:**
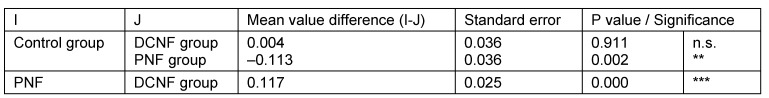
Pairwise comparison of mean value differences of sella turcica area with respect to main diagnostic groups

**Table 20:**
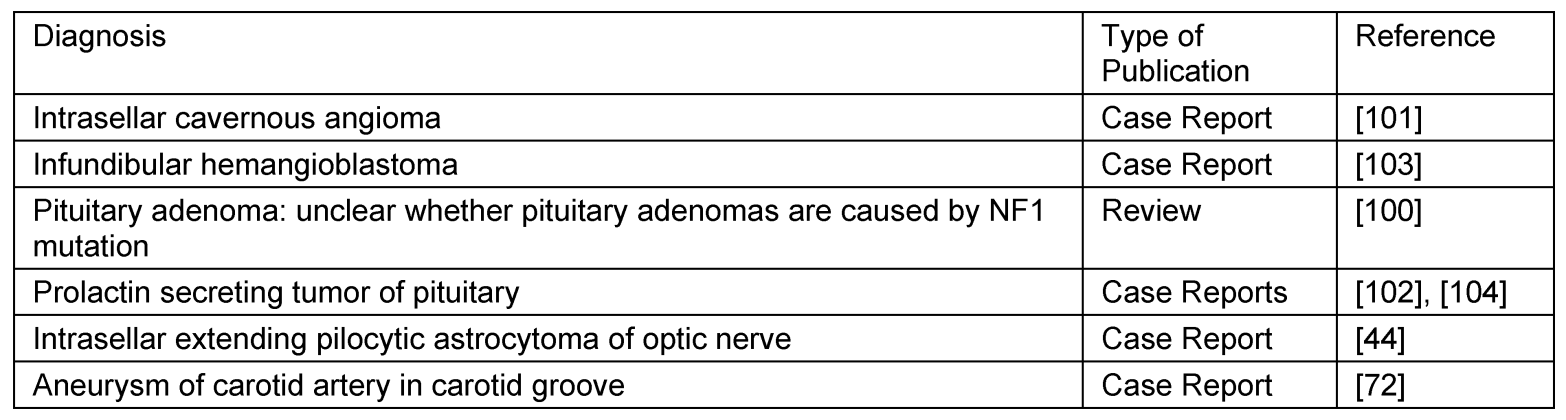
Pathologies adjacent to the sella region have to be considered in evaluation of sella turcica morphology of NF1 patients. Selected reports from the literature.

**Table 21:**
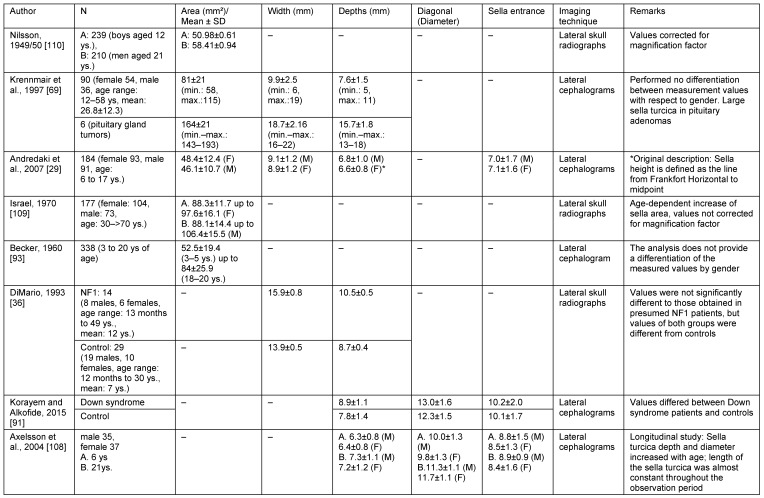
Selected studies providing data of sella turcica morphology (lines, area) on radiographs
